# OneStopRNAseq: A Web Application for Comprehensive and Efficient Analyses of RNA-Seq Data

**DOI:** 10.3390/genes11101165

**Published:** 2020-10-02

**Authors:** Rui Li, Kai Hu, Haibo Liu, Michael R. Green, Lihua Julie Zhu

**Affiliations:** 1 Department of Molecular, Cell and Cancer Biology, University of Massachusetts Medical School, 364 Plantation Street, Worcester, MA 01605, USA; rui.li@umassmed.edu (R.L.); kai.hu@umassmed.edu (K.H.); haibo.liu@umassmed.edu (H.L.); michael.green@umassmed.edu (M.R.G.); 2 Program in Molecular Medicine, Program in Bioinformatics and Integrative Biology, University of Massachusetts Medical School, Worcester, MA 01605, USA

**Keywords:** RNA-seq, workflow, pipeline, web application, quality control, visualization, differential gene expression, alternative-splicing analysis, allele–specific expression quantification, differential transposable element expression analysis, differential exon usage, GSEA

## Abstract

Over the past decade, a large amount of RNA sequencing (RNA-seq) data were deposited in public repositories, and more are being produced at an unprecedented rate. However, there are few open source tools with point-and-click interfaces that are versatile and offer streamlined comprehensive analysis of RNA-seq datasets. To maximize the capitalization of these vast public resources and facilitate the analysis of RNA-seq data by biologists, we developed a web application called OneStopRNAseq for the one-stop analysis of RNA-seq data. OneStopRNAseq has user-friendly interfaces and offers workflows for common types of RNA-seq data analyses, such as comprehensive data-quality control, differential analysis of gene expression, exon usage, alternative splicing, transposable element expression, allele-specific gene expression quantification, and gene set enrichment analysis. Users only need to select the desired analyses and genome build, and provide a Gene Expression Omnibus (GEO) accession number or Dropbox links to sequence files, alignment files, gene-expression-count tables, or rank files with the corresponding metadata. Our pipeline facilitates the comprehensive and efficient analysis of private and public RNA-seq data.

## 1. Introduction

The transcriptome is composed of diverse species of RNA, including protein-coding messenger RNA (mRNA) and noncoding RNA (ncRNA), and both are transcribed and expressed in a broad range of abundance in a given cell type [1]. mRNA is the essential intermediate in gene expression, bridging the genome to protein function [2]; ncRNAs can regulate gene expression by modulating chromatin formation and regulation, translation, macromolecule interactions, or even catalytic processes [3,4,5]. The transcriptome dynamically changes in response to internal and external cues; thus, it can be used as a proxy for gene-transcription activities [6,7], and abundance of gene end products on the bulk level under steady-state conditions [8,9,10]. With the development of numerous molecular techniques, the identification and quantification of transcriptome components has been one of the most convenient and informative avenues to understanding the molecular mechanisms of many biological processes and their regulation [2,11]. In particular, next-generation-sequencing (NGS) technologies have revolutionized the study of transcriptomes due to their single-base resolution, high sensitivity, high throughput, and broad dynamic range, and have dramatically decreased costs over the past decade [1,12,13].

First reported in 2008, RNA sequencing (RNA–seq) is a state-of-the-art method that characterizes the transcriptome by sequencing transcripts using NGS technologies [14]. Over the years, RNA–seq protocols have been improved to increase sensitivity, accuracy, and reproducibility, with reduced biases [13,15,16,17,18]. RNA–seq has been widely used to profile the changes of transcriptomes between conditions to understand the cause and effect of biological processes through differential gene-/transcript-/exon-expression analysis [19,20]. Beyond differential gene-expression analysis, RNA–seq can also be applied to achieve more detailed transcriptome characterization, including the analysis of alternative splicing (AS) and transposable-element (TE) expression, RNA modification and editing, and the identification of novel transcripts. Accordingly, RNA–seq has proven an ideal approach for novel transcriptome assembly, which is especially helpful for genome annotation for non-model organisms. As it is sequence-based, RNA–seq has also proven helpful in identifying expression genetic variants, for expression quantitative trait loci (eQTL) analysis, and even clinical diagnosis [19,21,22,23,24,25]. Furthermore, RNA-seq data play an important role in systems biology when integrated with other “omics”-scale data [26,27,28,29,30,31]. In summary, RNA–seq has been widely used in many fields, from basic research to clinical applications [32].

To date, hundreds of thousands of RNA-seq datasets and their metadata have been deposited to public data repositories such as NCBI GEO [33] and SRA [34], and data portals hosted by consortia, such as ENCODE (https://www.ncbi.nlm.nih.gov/geo/info/ENCODE.html), GTEx (https://www.gtexportal.org/home/datasets), and TCGA (https://isb-cancer-genomics-cloud.readthedocs.io/en/latest/sections/data/TCGA_top.html). As the cost of high-throughput sequencing continues to decrease, the amount of publicly available RNA-seq data continues to expand. 

Accompanying the large volume of RNA-seq data, a number of open source software packages were developed, from basic raw-read quality control (QC) to advanced pathway and network analysis (see review [35]). However, most of these open source tools are usually limited to a particular analysis step and have specific requirements on input types/formats. Users usually have to find multiple distinct packages and integrate them into a workflow to accomplish comprehensive RNA-seq data analysis. As a result, a certain level of programming skills is needed, which deters most biologists from analyzing RNA-seq data. 

A few graphical-user-interface (GUI)-based applications, such as Strand NGS (https://www.strand-ngs.com/), CLC Genomics Workbench (https://digitalinsights.qiagen.com), Lasergene Genomics (https://www.dnastar.com/software/genomics/), OmicsBox (https://www.biobam.com/omicsbox), Basepair (https://www.basepairtech.com/), and Partek Genomics Suite (https://www.partek.com/partek-genomics-suite/), have been commercialized to facilitate biologists analyzing sequencing data, but these commercial tools are usually very expensive. 

To meet the demands of many researchers without programming skills, dozens of free GUI- or web-interface-based workflows for RNA-seq data analysis were developed over the years. However, some of them suffer from a lack of maintenance and are outdated or even discontinued; others only have limited functionality. A full comparison of RNA-seq data-analysis workflows is shown in Supplementary Table S1. Well-maintained, fully featured, biologist-friendly analysis workflows are still needed. 

To maximize the capitalization of existing RNA-seq data, and enable biologists to analyze their own data and public RNA-seq datasets easily and rapidly, we developed web application OneStopRNAseq for the one-stop comprehensive analysis of RNA-seq data. It contains modules for read quality assessments (QA), read alignment, post-alignment RNA-seq-specific QA, count summarization, and differential gene expression (DGE), differential exon usage (DEU), and differential alternative splicing (DAS) analyses. It also supports differential transposable element expression (DTE) analysis, allele-specific gene expression (ASE) quantification, GO terms and KEGG pathway overrepresentation analysis, and MSigDB-based gene-set enrichment analysis (GSEA). In addition, OneStopRNAseq provides solutions for expression-count-table-based data analysis and visualization. 

Our workflow is biologist-oriented, with intuitive web interfaces for uploading data, and browsing and downloading results. We modularized the workflow implementation to enable streamlined analysis, easy maintenance, and feature expansion upon user feedback and requests. 

## 2. Materials and Methods

### 2.1. Implementation

OneStopRNAseq is implemented as a web application hosted by an Apache web server. A MySQL relational database is used at the back end, and the business/presentation layer is written in PHP. The Snakemake workflow-management system [36] was used to build the robust, reproducible, and scalable analysis pipeline. The common parameter settings for different types of analysis workflows were prepopulated, and some can be easily customized to meet users’ specific needs.

OneStopRNAseq employs the widely used FastQC [37] to check raw read quality, and MultiQC [38] to generate an integrated report. The workflow adopts STAR for read alignment [39]. Currently, RNA-seq data analyses based on human, mouse, yeast, fruit fly, zebrafish, and worm genomes are supported. However, other genomes can be easily added in response to users’ requests. Post-alignment RNA-seq quality control is performed using QoRTs [40] to output the most comprehensive visualization of quality metrics of RNA-seq data. The workflow uses featureCounts [41] to obtain a gene-level count table from BAM files, rMATS [42] for detecting DAS, DEXseq [43] for DEU analysis, SalmonTE [44] for TE expression quantification, DESeq2 [45] for DGE and DTE analysis, ASEReadCounter of GATK [46] for allele-specific expression quantification (ASE), and GSEA [47] for gene-set-enrichment analysis.

OneStopRNAseq is freely accessible to academic users at https://mccb.umassmed.edu/OneStopRNAseq. The Snakemake workflow is available for downloading or contributing at https://github.com/radio1988/OneStopRNAseq. 

### 2.2. RNA-Seq Data

To demonstrate the utility of our pipeline, we reanalyzed a public RNA-seq dataset (GSE151286) from the GEO repository [48]. Briefly, RNA-seq data consisted of eight human lung-tumor cell line NCI-H526 samples with two biological replicates for each treatment-by-time combination. Cells were treated with either DMSO vehicle control (CK) or 0.5 µM of USP7 inhibitor USP7-797 (USP7797) for 24 or 48 h. Data were generated using unstranded RNA-seq libraries and sequenced on an Illumina NovaSeq platform in 2 × 100 bp paired-end mode.

## 3. Results

### 3.1. Functionality Summary of the OneStopRNAseq Application

OneStopRNAseq (https://mccb.umassmed.edu/OneStopRNAseq) is an easy-to-use web application designed for the comprehensive analyses of RNA-seq data for both biologists and bioinformaticians. In order to simplify and streamline RNA-seq analyses, we integrated a set of widely used analysis components into our pipeline, including DGE, DEU, DAS, GSEA, and DTE analyses, and ASE quantification.

**Figure 1:**
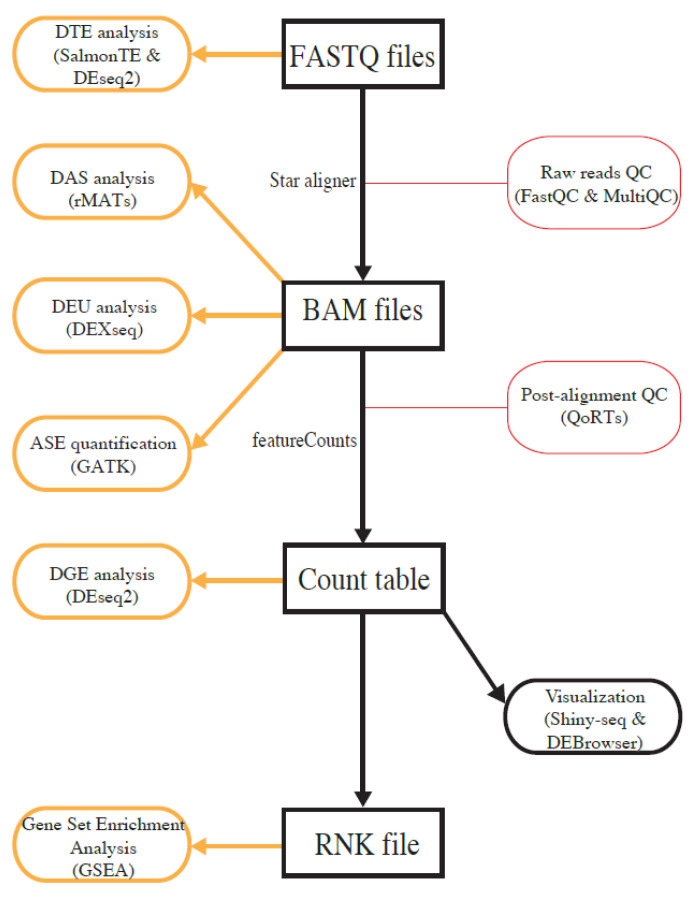
Overview of analysis workflows implemented in OneStopRNAseq. Software packages for each analysis task are shown in brown round-cornered rectangles enclosed by round brackets and along vertical black arrows.

To make it convenient for users, we implemented four major analysis paths with different analysis entry points, i.e., raw FASTQ files, binary alignment map (BAM) files, gene-expression count table files, and rank files (Figure 1). Users can select the analysis path on the basis of the type of available data and types of desired analysis. If users start the analysis with FASTQ files, all types of analyses are performed, although ASE quantification requires users to provide an additional variant-call-format (VCF) file containing genotype information. To perform DGE analysis and GSEA, users can also start with a gene-expression count table. To merely run GSEA, users only need to upload a ranked gene list. A detailed user guide is included as a supplementary file, and it is also available under the Help menu at https://mccb.umassmed.edu/OneStopRNAseq, which will be updated when additional features are added. 

Private RNA-seq data are stored locally or more commonly in commercial cloud-storage spaces such as Dropbox, OneDrive, Google Drive, Box, and pCloud, which provide high data security, simple data sharing, and easy data management. Among them, Dropbox has emerged as a popular cloud-storage space for data sharing (https://www.pcmag.com/picks/the-best-cloud-storage-and-file-sharing-services). To use OneStopRNAseq to analyze RNA-seq data in Dropbox, users can simply provide shared Dropbox links to their data and sample information (metadata) through the web interface or upload an Excel spreadsheet with the required metadata, and specify the conditions to compare. 

OneStopRNAseq is also optimized for analyzing public datasets. To analyze RNA-seq data in the GEO database, users only need to input accession number(s) of interest, verify or modify the automatically retrieved metadata, and specify the conditions to compare.

Additionally, we integrated DEBrowser [49] and Shiny-Seq [50] for the interactive exploratory analysis of gene-expression data and differential gene-expression analysis. With both Shiny apps, users can start with gene-expression count tables, perform exploratory data analysis with boxplots and principal-component-analysis (PCA) plots, batch-effect correction, DGE, gene coexpression analysis using WGCNA [51], over-representation analysis of GO terms, KEGG pathways, and disease ontology terms, and GSEA. Alternatively, users can start with FASTQ files and perform Kallisto-based pseudoalignment [52] to quickly obtain a gene-expression count or transcripts-per-kilobase-million (TPM) [53] tables and perform all interactive analyses as above using Shiny-Seq. 

At the back end, the Snakemake workflow management system is used for reproducible and scalable data analyses. The workflow is open source, so users can find out exactly which analyses are being performed and which parameters are being used. Bioinformaticians and power users can also download workflows and run the analysis in their own Linux workstation or high-performance-computing (HPC) system, with most of the package installed automatically with Anaconda (https://www.anaconda.com/) or wrapped in singularity (https://singularity.lbl.gov/) images. To download the workflow, please visit our GitHub repository (https://github.com/radio1988/OneStopRNAseq).

### 3.2. Case Study Validating OneStopRNAseq Application Functionalities

We reanalyzed a recently published RNA-seq dataset GSE151286 [48] to illustrate the utility of our OneStopRNAseq application. All analysis modules except for allele-specific expression quantification were performed using the software and parameter settings listed in Supplementary Table S2, which is also available under the About menu at https://mccb.umassmed.edu/OneStopRNAseq.

**Figure 2:**
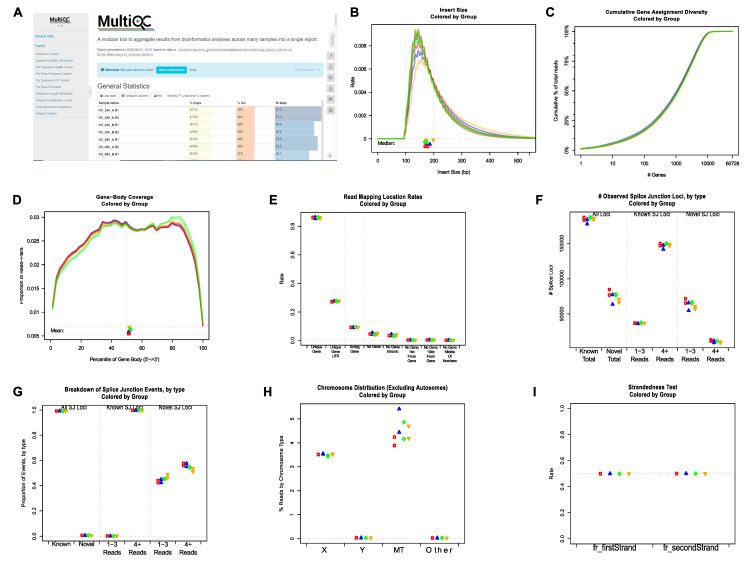
Representative plots showing quality control results generated by FastQC/MultiQC and QoRTs. (**A**) Top part of the HTML report generated using MultiQC by integrating the individual QC report outputted by FastQC. (B-I) Representative post-alignment QC plots generated by QoRTs. (**B**) Distributions of RNA-seq library insert sizes. (**C**) Cumulative gene assignment diversity. (**D**) Read coverage along gene body. (**E**) Percentage of reads mapped to different genomic regions. (**F**) Numbers of known and novel splicing junctions. (**G**) Percentages of known and novel splicing junctions. (**H**) Percentages of reads mapped to non-autosomes. (**I**) Strandedness of RNA-seq libraries.

First, the sequencing quality of the raw reads of individual FASTQ files was checked using FastQC [37]. The final all-in-one quality-control report was generated using MultiQC [38]. Representative plots showing multiple quality metrics of raw sequencing data are shown in Supplementary Figure S1 and Figure 2A. To perform RNA-seq data-specific quality control, BAM files produced by STAR [39] were analyzed using QoRTs [40]. Examples of relevant plots showing RNA-seq data quality are shown in Figure 2B–I. Both FastQC and QoRTs analyses demonstrated that the RNA-seq data were of high quality. 

**Figure 3:**
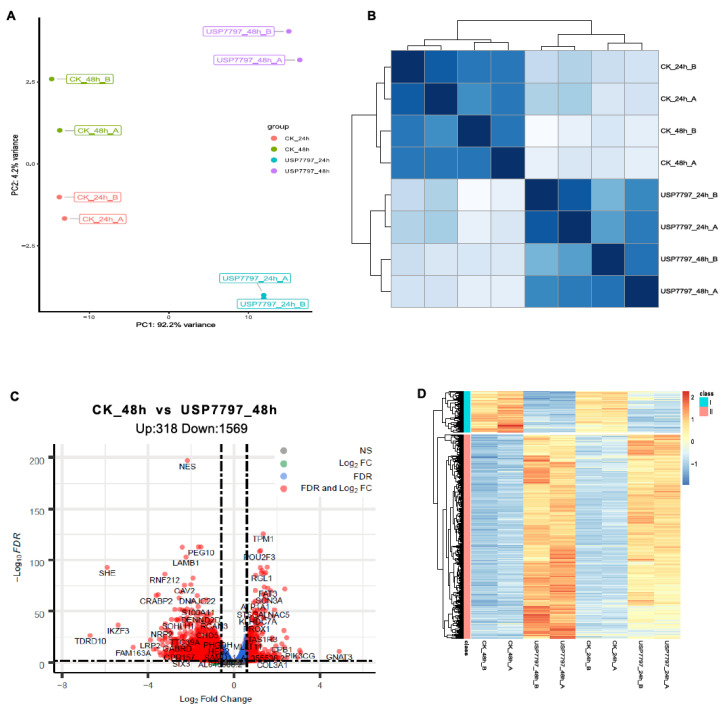
Exploratory and differential expression analyses of RNA-seq data. (**A**) Principal-component analysis of sample relationship. (**B**) Poisson distance plot of sample dissimilarities in terms of transcriptomic profiles. (**C**) Volcano plot of shrunken log_2_ (fold change) and –log_10_FDR of all tested genes between vehicle-control (CK) samples and samples treated with USP7797 at 48 h post treatment. Genes with|log_2_(fold change)| > log_2_(1.5) and FDR < 0.05 (significantly differentially expressed genes) are in red, genes with|log_2_(fold change)| > log_2_(1.5) but FDR ≥ 0.05 are in green, genes with|log_2_(fold change)| ≤ log_2_(1.5) but FDR < 0.05 are in blue, and the rest are in gray. (**D**) Heatmap showing significantly differentially expressed genes which are represented by red dots in (**C**).

**Figure 4:**
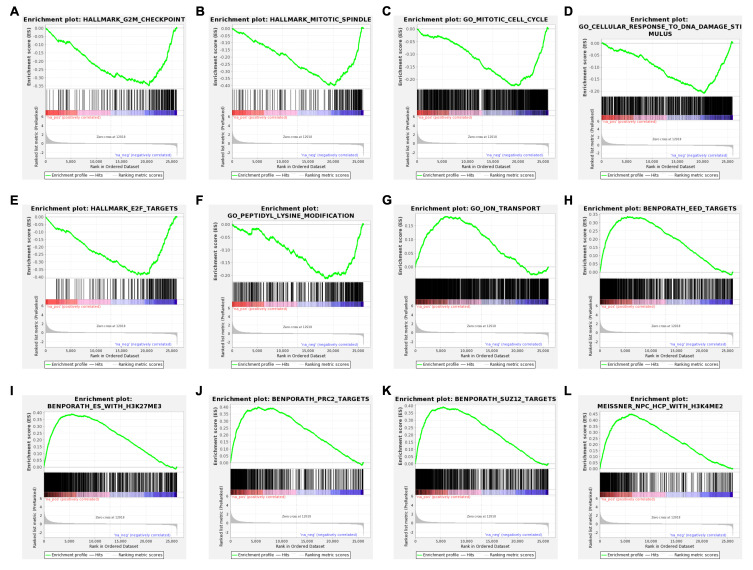
Enrichment plots showing significantly enriched gene sets (FDR < 0.05) in ranked gene list for samples treated with USP7797 for 24 h compared to those treated with DMSO vehicle control for 24 h. Running enrichment scores (ES) and locations of the members of the gene set in the ranked list of genes are shown for a dozen top representative molecular signatures from the Molecular Signatures Database (MSigDB) at https://www.gsea-msigdb.org/gsea/msigdb. H: hallmark gene sets are shown in (**A**,**B**,**E**). C5: ontology gene sets are shown in (**C**,**D**,**F**,**G**). C2: curated gene sets are shown in (**H**–**L**). (**A**–**F**) show negative ES where the leading edge subset appears in the ranked list subsequent to the valley score. (**G**–**L**) show positive ES where the leading edge subset appears in the ranked list prior to the peak score.

A gene-level read-count table was generated using featureCounts [41]. Principal component analysis (PCA) and Poisson distance plots (Figure 3A,B) demonstrated that gene expression profiles of USP7797-treated samples were clearly different from those of CK samples. Differentially expressed genes were identified using DESeq2 by testing three contrasts: CK_24h—USP7797_24h, CK_48h—USP7797_48h, and (USP7797_48h—USP7797_24 h) – (CK_48h—CK_24h). The volcano plot (Figure 3C) and heatmap (Figure 3D) display the differentially expressed genes between CK samples and those treated with USP7797 at 48 h post treatment. MSigDB-based gene-set enrichment analysis (GSEA) verified the results reported by the original publication [48]. For example, USP7797 treatment downregulated the expression of many genes involved in the cell cycle (G2M checkpoint, mitotic spindle, mitotic cell cycle; Figure 4A–C). USP7797 treatment also upregulated the expression of genes that are normally silenced by polycomb repressive complex 2 (PRC2) (Figure 4H–L [54]). Additionally, USP7797 treatment downregulated the expression of genes responsive to DNA damage stimulus (Figure 4D), and those facilitating histone acetylation and ubiquitination (Figure 4F and Supplementary Table S3. The expression of many E2F targets was downregulated, and many genes involved in ion transportation were upregulated by USP7797 (Figure 4F,G). Gene ontology–biological process terms (protein monoubiquitination, protein polyubiquitination and histone deubiquitination) and associated gene sets were enriched among downregulated genes in response to USP7797 treatment (Supplementary Table S3). These gene-set-enrichment analysis results are consistent with the role of USP7797 as a small-molecule inhibitor of the ubiquitin-specific peptidase (USP7) that cleaves ubiquitin from its substrates [48,55]. 

**Figure 5:**
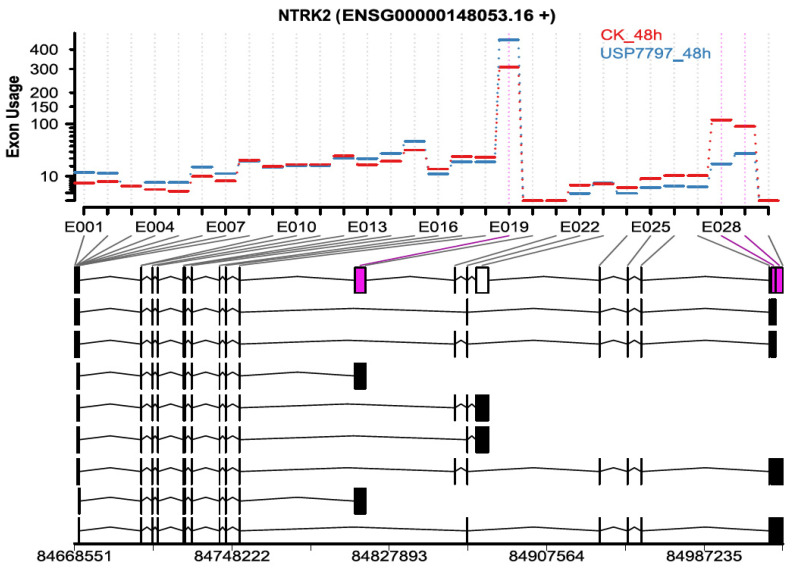
Representative plot showing differential exon usage between samples treated with USP7797 for 48 h and those treated with DMSO vehicle for 48 h. Significantly differentially used exons 19, 28, and 29 are in purple.

In addition to differential gene-expression analysis, as performed by Ohol et al. [48], we performed differential exon-usage and alternative-splicing analyses using the OneStopRNAseq application. We identified 819 and 4666 exons of differential usage (|log_2_(fold change)| ≥ log_2_(1.5) and FDR < 0.05) for contrasts USP7797_24h—CK_24h and USP7797_48h—CK_48h, respectively. Only two exons showed significant time-by-treatment interaction effect on differential exon usage (FDR < 0.05). Figure 5 shows one of the top differentially used exons between the sample treated with USP7797 and the CK at 48 h post treatment. We also identified a small number of differential alternative splicing events (see Supplementary Table S4); thus, OneStopRNAseq facilitates the simultaneous identification of alternative-splicing events, differentially used exons, and differentially expressed genes, which can be used to generate potential hypothesis for further investigation.

### 3.3. Runtime of OneStopRNAseq Application

**Figure 6:**
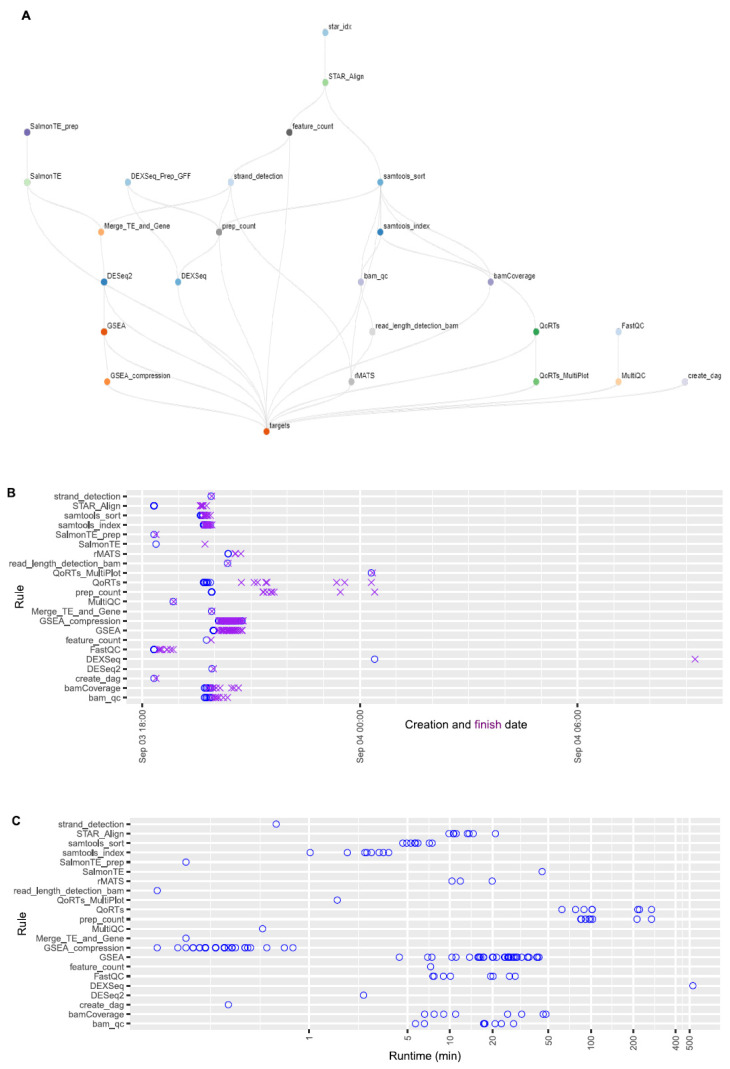
The structure of the OneStopRNAseq workflow and runtime statistics of each job. (**A**) A directional acyclic graph (DAG) showing the structure of the workflow. Jobs at lower levels depend on connected jobs at higher level and the workflow is executed from the top to the bottom following the specified job dependencies. (**B**) Creation and finish dates of each job. Blue circles indicate job creation dates and purple crosses show job finish dates. (**C**) Runtime of each job in minutes.

Estimating expected runtimes for computational pipelines can be challenging, as they are influenced by multiple variables, including the size of the input-data files, the number of available numbers of central processing units (CPU), and the amount of random-access memory (RAM) per CPU, as well as the potential number of parallel threads used for each job. Overall wall-clock times depend on the availability of the computing resources when jobs are submitted, job dependency, and the actual runtime of each job. Figure 6A shows the topological structure of the workflow. Here, we provide the job creation, finish timeline (Figure 6B), and runtime of each job for the case study given the computing resources specified by the current implementation (Supplementary Table S2). On the basis of Figure 6B, overall wall-clock time was determined by DEXseq jobs, which were created at a later time because they depended on prep_count jobs. Task DEXseq had the longest runtime (530 minutes) due to the large number of exons to be analyzed, followed by tasks prep_count and QoRTs (Figure 6C). Overall, the whole analysis process was finished within 15 h.

## 4. Discussion

RNA-seq has become a widely used technology in many fields, including genomics and clinical diagnostics, but only differential gene-expression analysis has been performed for the majority of RNA-seq experiments, partially due to the lack of comprehensive RNA-seq analysis pipelines. To fill this gap, we developed an easy-to-use web application, OneStopRNAseq, which enables the comprehensive analyses of both private and public RNA-seq data.

Compared to most existing RNA-seq data-analysis pipelines, OneStopRNAseq integrates the largest number of analysis modules (Supplementary Table S2). Each analysis module leverages one or more widely accepted tools, chosen on the basis of current best practices for RNA-seq data analysis [19,20]. 

For DGE analysis, in addition to output generated from DESeq2 analysis, our pipeline automatically generates sample-labeled PCA and Poisson distance plots (robust to uncertainty in lowly expression genes). These two plots are useful for identifying issues with the samples such as library preparation and visualizing global changes among different samples or experiment conditions. Additional plots include gene-symbol-annotated volcano plots by EnhancedVolcano (https://github.com/kevinblighe/EnhancedVolcano) and heatmaps for significant differentially expressed genes by pheatmap (https://github.com/raivokolde/pheatmap). Furthermore, our pipeline also outputs results from GSEA analysis, which takes a rank-ordered gene list without requiring users to select a subset of genes on the basis of an arbitrary cut-off, and can take differential expression analysis from gene level to gene-set, pathway, and GO-term level.

The vast majority of genes undergo some level of AS, which contributes to protein diversity, and they regulate many biological processes [56]. We incorporated rMATS, a popular event-based AS analysis tool [42]. Alternative-splicing events identified by rMATS include skipped exon (SE), alternative 5’ splice site (A5SS), alternative 3’ splice site (A3SS), mutually exclusive exons (MXE), and retained intron (RI). Sometimes, sequencing depth is not enough for reliable event-based DAS analysis, but enough for DEU analysis; therefore, we also incorporated DEXSeq, a popular tool for DEU analysis, into our pipeline [57]. 

Allele-specific gene expression plays an important role in tumor initiation and progression [58]. We incorporated ASEReadCounter from GATK [59] into our pipeline for users to obtain allele-specific expression quantification results when single nucleotide polymorphism (SNP) information of individuals or strains (e.g., mouse strains) in the VCF format is provided. The output format is compatible with Mamba [58], which is a downstream tool for differential ASE analysis.

Besides integrating existing tools, we also achieved some innovations. For instance, we combined DTE and DGE analyses to improve the robustness and sensitivity of DTE analysis. TE is generally ignored in most standard RNA-seq analysis pipelines [60], and most genome annotation files do not have TE entries. We incorporated SalmonTE [44], the fastest tool in DTE analysis, into our analysis pipeline; however, the SalmonTE analysis pipeline performs DTE on TE expression quantification tables with DESeq2 [45] without considering mRNA expression. There are two problems with this approach. First, there are only a few hundred or fewer TEs in most species, and normalization with such a small number of genes is not robust to variances in TE expression abundance. Second, DESeq2’s median of ratio normalization assumes that the majority of the genes do not differ in expression between groups. Analyzing TE expression alone leads to false positives/negatives when most TEs are up- or downregulated. To overcome this, we combined the TE expression table with the standard gene-expression table to obtain more robust results. This approach has proven useful in our own data analysis (unpublished results) and is also implemented in the workflow of TEsmall (http://hammelllab.labsites.cshl.edu/software/#TEsmall). 

Unlike the majority of existing RNA-seq analysis pipelines, OneStopRNAseq can handle complicated designs with more than two groups. For example, with a randomized complete block design, users can input the factor of interest as GROUP_LABEL and blocking factors that are not of research interest under BATCH_LABEL. With the factorial design, users can enter GROUP_LABEL as the concatenation of labels from different factors. For example, with a two-by-two factorial design consisting of two treatments (CK and USP7797) and two time points (24 and 48 h) in the aforementioned case study, users can enter the GROUP_LABEL as CK_24h, CK_48h, USP7797_24h, and USP7797_48h. Users can specify any comparisons or contrasts, such as the main effect of treatment and time, any pairwise comparisons, and the differential effect of treatment at different time points. Detailed instructions on how to specify various types of contrasts for DGE and DAS analysis are included in the user guide as a supplementary file, and are available under the Help menu of https://mccb.umassmed.edu/OneStopRNAseq. Under the Help menu, descriptions of the output files and a template for writing the analysis method using OneStopRNAseq are also provided.

OneStopRNAseq was developed not only for analyzing users’ own data, but it is also convenient for biologists and bioinformaticians who analyze public datasets. Our pipeline facilitates the comprehensive and efficient analysis of RNA-seq data. To further increase OneStopRNAseq’s appeal to a broader user community, we plan to integrate additional applications such as genome-guided transcriptome assembly to facilitate the assessment of completeness of transcriptome assembly and the identification of novel isoforms for more accurate DEU and DAS analysis. In addition, we also plan to integrate ensemble gene set enrichment analysis tools such as EGSEA [61] and alternative DGE analysis tools such as edgeR [62,63] to facilitate tool comparisons and novel tool development.

## Supplementary Materials

The following are available online at https://www.mdpi.com/2073-4425/11/10/1165/s1, Figure S1. Plots showing raw sequencing QC. Data quality of individual RNA-seq files was analyzed by FastQC. MultiQC was used to generate an all-in-one HTML reports. Shown here are plots from the final HTML report. (A) Scatterplot showing average GC% and total read numbers. (B) The numbers of unique and duplicate reads. (C) Per-base mean read quality scores. (D) Per-sequence quality Scores. (E) Per-sequence GC contents. (F) Sequence duplication rate. (G) Overrepresented sequences. (H) Adaptor contents. Table S1. Comparison of RNA-seq data analysis workflows. Table S2. Software and parameter settings used by the OneStopRNAseq workflow. Table S3. Enriched Gene Ontology (GO) terms related to protein ubiquitination among down-regulated genes by USP7797 treatment compared to DMSO vehicle control. Table S4. Summary of differential alternative splicing events identified by rMATS.
