# Supplementary material for: OneStopRNAseq: A Web Application for Comprehensive and Efficient Analyses of RNA-Seq Data
Source: Genes (Basel). 2020 Oct 2;11(10):1165. doi: 10.3390/genes11101165 (PMC7650687; doi:10.3390/genes11101165)
Supplement: Supplementary file 1 [file genes-11-01165-s001.zip › SupplementaryFiles/Table S2.Software and parameter settings used by the OneStopRNAseq workflow.docx]

**Supplementary Table2.**  **Software and parameter settings used by OneStopRNAseq v1.0.0**

| Tools | Version | Parameters for users  (and default values) | Parameters | Link (reference) |
| --- | --- | --- | --- | --- |
| FastQC | 0.11.5 | None | Default | <https://www.bioinformatics.babraham.ac.uk/projects/fastqc/> |
| MultiQC | 1.6 | None | Default | <https://multiqc.info>  (Ewels et al., 2016) |
| STAR | 2.7.5a | Reference Genome^1^ | STAR --runThreadN {threads}  --genomeDir {INDEX}  --sjdbGTFfile {gtf}  --readFilesCommand zcat  --readFilesIn {reads}  --outFileNamePrefix {name}  --outFilterType BySJout  --outMultimapperOrder Random  --outFilterMultimapNmax 200  --alignSJoverhangMin 8  --alignSJDBoverhangMin 3  --outFilterMismatchNmax 999  --outFilterMismatchNoverReadLmax 0.05  --alignIntronMin 20  --alignIntronMax 1000000  --outFilterIntronMotifs RemoveNoncanonicalUnannotated  --outSAMstrandField None  --outSAMtype BAM Unsorted  --quantMode GeneCounts  --outReadsUnmapped Fastx | <https://github.com/alexdobin/STAR>  (Dobin et al., 2013) |
| QoRTs | 1.3.6 | None | Default | <https://hartleys.github.io/QoRTs/>  (Hartley & Mullikin, 2015) |
| Samtools | 1.9 | None | Default with more RAM and threads | <http://www.htslib.org/>  (Li et al., 2009) |
| featureCounts | 2.0.0 | strandness (Auto)  MODE^2^ (strict) | MODE strict paired-end:  -Q 20 --minOverlap 1  --fracOverlap 0 -p -B -C  MODE liberal paired-end:  -M --primary -Q 0  --minOverlap 1 --fracOverlap 0 -p  MODE strict single-end:  -Q 20 --minOverlap 1  --fracOverlap 0  MODE liberal single-end:  -M --primary -Q 0  --minOverlap 1 --fracOverlap 0 | <http://bioinf.wehi.edu.au/featureCounts/>  (Liao et al., 2014) |
| SalmonTE | 0.4 | None | python SalmonTE.py quant  --reference={ref} --exprtype=count  --num_threads={threads}  {read1} {read2} | <https://github.com/LiuzLab/SalmonTE>  (Jeong et al., 2018) |
| DESeq2 | 1.28.1 | MAX_FDR (0.05)  MIN_LFC (0.585)  cooksCutoff ^3^ (TRUE)  independentFiltering^4^ (FALSE) | With batch effect:  design = ~ 0 + group + batch  Without batch effect:  design = ~ 0 + group | <https://bioconductor.org/packages/release/bioc/html/DESeq2.html>  (Love et al., 2014) |
| DEXSeq | 1.34.0 | None | default | <https://bioconductor.org/packages/release/bioc/html/DEXSeq.html>  (Anders et al., 2012) |
| rMATS | 4.1.0 | None | python rmats.py  --b1 b1.txt --b2 b2.txt  --gtf {gtf} -t {type}  --readLength {length}  --variable-read-length  --libType {strandness}  --nthread {threads}  --tstat {threads}  --cstat 0.2  --od output --tmp tmp | <http://rnaseq-mats.sourceforge.net/rmats4.1.0/>  (Shen et al., 2014) |
| GSEA | 4.0.3 | NPLOTS^5^ (100) | gsea-cli.sh GSEAPreranked  -gmx {db} -rpt_label {db}  -rnk {rnk}  -norm meandiv -nperm 1000  -scoring_scheme classic  -create_svgs {svg}  -make_sets true  -rnd_seed timestamp  -zip_report false  -set_max 15000 -set_min 15  -plot_top_x {GSEA_NPLOTS} -out ./gsea/{contrast} | <https://www.gsea-msigdb.org/gsea/index.jsp>  (Subramanian et al., 2005) |
| deepTools | 3.1.3 | MODE (strict) | MODE strict:  bamCoverage --bam {input.bam}  -o {output}  --numberOfProcessors {threads}  --outFileFormat bigwig  --normalizeUsing CPM --binSize 10  --minMappingQuality 20  MODE liberal:  bamCoverage --bam {input.bam}  -o {output}  --numberOfProcessors {threads}  --outFileFormat bigwig  --normalizeUsing CPM --binSize 10 | <https://deeptools.readthedocs.io/en/develop/index.html>  (Ram et al., 2016) |

1. Available reference genomes and annotations for users to select.

| Species | Genome | Annotation |
| --- | --- | --- |
| Human | hg38 | gencode.v34.primary_assembly |
| Mouse | mm10 | gencode.vM25.primary_assembly |
| Worm (*C. elegans*) | WBcel235 | WBcel235.90 |
| Yeast (*S. cerevisiae*) | R64-1-1 | R64-1-1.90 |
| Fruit fly (*D. melanogaster*) | BDGP6 | BDGP6.22.96 |
| Zebra fish (*D. rerio*) | danRer11 | V4.3.2 (Lawson et al., 2020) |

1. The MODE parameter in featureCounts: default to strict. The corresponding parameter in the web interface is “ Include only uniquely mapped reads (Yes)”

strict: only uniquely mapped reads are included in the gene quantification.

liberal: reads that are mapped equally well to multiple locations on the genome will also be quantified and assigned to one of the locations randomly. This setting is useful if you know some of the genes of your interest have multiple copies on the genome, e.g. histone genes

1. The cooksCutoff parameter in DEseq2: default to TRUE

TRUE: The *p* values and adjusted *p* values are set to NA for genes that contain a Cook’s distance above a cutoff for samples which have at least three replicates. Cook’s distance measures the magnitude of the influence of a single sample on the fitted coefficients for a gene, and a large value of Cook’s distance indicates an outlier count. For more detailed information, please refer to the section “Approach to count outliers“ at <https://bioconductor.org/packages/release/bioc/vignettes/DESeq2/inst/doc/DESeq2.html>

FALSE: No genes will be flagged with NA *p* values or adjusted *p* values because of a large Cook’s distance.

1. The independentFilter parameter in DESeq2: default to FALSE

TURE: Exclude those tests that have very little chance of showing significant evidence using test statistic independent filtering statistics such as the mean of normalized counts. This will result in increased detection power at the same experiment-wide type I error. For more detailed information, please refer to <https://bioconductor.org/packages/release/bioc/vignettes/DESeq2/inst/doc/DESeq2.html#independent-filtering-of-results>.

FALSE: No independent filtering will be performed.

1. The NPLOTS parameter in GSEA: default to 100. The corresponding parameter in the web interface is “Please specify the number of top gene sets to be plotted (100)”

# Number of top gene sets (ranked by p-value) for which the enrichment plots will be created. For more information on available gene sets, please refer to <https://www.gsea-msigdb.org/gsea/msigdb>. An example of enrichment plot is available at <https://www.gsea-msigdb.org/gsea/doc/GSEAUserGuideTEXT.fld/image009.jpg>. To properly interpret the GSEA results, please refer to the section “Interpreting GSEA Results“ at https://www.gsea-msigdb.org/gsea/doc/GSEAUserGuideFrame.html.

**References:**

Anders, S., Reyes, A., & Huber, W. (2012). Detecting differential usage of exons from RNA-seq data. *Genome Research*, *22*(10), 2008–2017. https://doi.org/10.1101/gr.133744.111

Dobin, A., Davis, C. A., Schlesinger, F., Drenkow, J., Zaleski, C., Jha, S., Batut, P., Chaisson, M., & Gingeras, T. R. (2013). STAR: Ultrafast universal RNA-seq aligner. *Bioinformatics*. https://doi.org/10.1093/bioinformatics/bts635

Ewels, P., Magnusson, M., Lundin, S., & Käller, M. (2016). MultiQC: Summarize analysis results for multiple tools and samples in a single report. *Bioinformatics*. https://doi.org/10.1093/bioinformatics/btw354

Hartley, S. W., & Mullikin, J. C. (2015). QoRTs: A comprehensive toolset for quality control and data processing of RNA-Seq experiments. *BMC Bioinformatics*. https://doi.org/10.1186/s12859-015-0670-5

Jeong, H. H., Yalamanchili, H. K., Guo, C., Shulman, J. M., & Liu, Z. (2018). An ultra-fast and scalable quantification pipeline for transposable elements from next generation sequencing data. *Pacific Symposium on Biocomputing*, *0*(212669), 168–179. https://doi.org/10.1142/9789813235533_0016

Lawson, N. D., Li, R., Shin, M., Grosse, A., Yukselen, O., Stone, O. A., Kucukural, A., & Zhu, L. (2020). An improved zebrafish transcriptome annotation for sensitive and comprehensive detection of cell type-specific genes. *ELife*. https://doi.org/10.7554/eLife.55792

Li, H., Handsaker, B., Wysoker, A., Fennell, T., Ruan, J., Homer, N., Marth, G., Abecasis, G., & Durbin, R. (2009). The Sequence Alignment/Map format and SAMtools. *Bioinformatics*. https://doi.org/10.1093/bioinformatics/btp352

Liao, Y., Smyth, G. K., & Shi, W. (2014). FeatureCounts: An efficient general purpose program for assigning sequence reads to genomic features. *Bioinformatics*. https://doi.org/10.1093/bioinformatics/btt656

Love, M. I., Huber, W., Anders, S., Love, M. I., Huber, W., & Anders, S. (2014). *Moderated estimation of fold change and dispersion for RNA-Seq data with DESeq2 Moderated estimation of fold change and dispersion for RNA-Seq data with DESeq2*.

Ram, F., Ryan, D. P., Bhardwaj, V., Kilpert, F., Richter, A. S., Heyne, S., Friederike, D., & Manke, T. (2016). *deepTools2 : a next generation web server for deep-sequencing data analysis*. *44*(April), 160–165. https://doi.org/10.1093/nar/gkw257

Shen, S., Park, J. W., Lu, Z. X., Lin, L., Henry, M. D., Wu, Y. N., Zhou, Q., & Xing, Y. (2014). rMATS: Robust and flexible detection of differential alternative splicing from replicate RNA-Seq data. *Proceedings of the National Academy of Sciences of the United States of America*. https://doi.org/10.1073/pnas.1419161111

Subramanian, A., Tamayo, P., Mootha, V. K., Mukherjee, S., Ebert, B. L., Gillette, M. A., Paulovich, A., Pomeroy, S. L., Golub, T. R., Lander, E. S., & Mesirov, J. P. (2005). Gene set enrichment analysis: A knowledge-based approach for interpreting genome-wide expression profiles. *Proceedings of the National Academy of Sciences*. https://doi.org/10.1073/pnas.0506580102
