# Supplementary material for: OneStopRNAseq: A Web Application for Comprehensive and Efficient Analyses of RNA-Seq Data
Source: Genes (Basel). 2020 Oct 2;11(10):1165. doi: 10.3390/genes11101165 (PMC7650687; doi:10.3390/genes11101165)
Supplement: Supplementary file 1 [file genes-11-01165-s001.zip › SupplementaryFiles/UserGuideOneStopRNAseq.docx]

User’s Guide for OneStopRNAseq

**Author List and Affiliations**

Rui Li, Department of Molecular, Cell and Cancer Biology, University of Massachusetts Medical School, 364 Plantation Street, Worcester, MA 01605, USA. Rui.Li@umassmed.edu

Kai Hu, Department of Molecular, Cell and Cancer Biology, University of Massachusetts Medical School, 364 Plantation Street, Worcester, MA 01605, USA. [Kai.Hu@umassmed.edu](mailto:Kai.Hu@umassmed.edu)

Haibo Liu, Department of Molecular, Cell and Cancer Biology, University of Massachusetts Medical School, 364 Plantation Street, Worcester, MA 01605, USA. [Haibo.Liu@umassmed.edu](mailto:Haibo.Liu@umassmed.edu)

Michael R. Green, Department of Molecular, Cell and Cancer Biology, University of Massachusetts Medical School, 364 Plantation Street, Worcester, MA 01605, USA. michael.green@umassmed.edu

Corresponding author: Lihua Julie Zhu, Department of Molecular, Cell and Cancer Biology, Department of Molecular Medicine, Program in Bioinformatics and Integrative Biology, University of Massachusetts Medical School, 364 Plantation Street, Worcester, MA 01655, USA. [Julie.Zhu@umassmed.edu](mailto:Julie.Zhu@umassmed.edu)

RL, KH, and HL contributed equally to this work.

**Overview**

_­_ OneStopRNAseq (<https://mccb.umassmed.edu/OneStopRNAseq>) is an easy to use web application designed for comprehensive analyses of RNA-seq data for biologists and bioinformaticians. In order to simplify and streamline RNA-seq analysis, we integrated many widely used analysis components into our pipeline, including differential gene expression (DGE) analysis, differential exon usage (DEU) analysis, differential alternative splicing (DAS) analysis, gene set enrichment analysis (GSEA), differential transposon element (DTE) analysis, and allele-specific expression (ASE) quantification. To be flexible and shorten analysis time, OneStopRNAseq provides multiple analysis entry points, i.e., users can start their analyses with raw FASTQ files, aligned BAM files, gene count table files or rank files according to the analysis goals and available files as shown in the right panel of Figure 1. In summary, users can simply put their data in Dropbox, provide Dropbox download links and sample information (metadata) through the website, select analyses of interest, and comparisons/contrasts of interest, submit the job, and get analysis results. Data uploading is carried out in batch via Wget and the users do not have to upload one file at a time via the website to avoid any interruption by unstable internet connection and long wait time for the users. Alternatively, users can input GEO accession number and the associated metadata will be automatically retrieved from the database. After users review and verify associated metadata, raw data will be downloaded and extracted using prefetch and fastq-dump automatically. Therefore, OneStopRNAseq is developed not only for analyzing your own data but also convenient for analyzing public datasets.

In the following section, we provide more details on how to use our web application. We will keep updating the user’s guide with new functionalities added. For the most recent version, we encourage you to visit our web site.

**Web interface**

**Figure 1** shows the homepage of our web application. We listed the currently supported analyses on the left panel where you can hover over each item to view the software packages that are being used. The main workflow is illustrated on the right panel where all of the black boxes (and round boxes) are “clickable”. The clickable boxes are possible choices of entry points to the pipeline. To start with “FASTQ” or “BAM” files, you have to register yourself by filling in your email and other information in the registration form, whereas no registration is needed to start the analysis from “Count table”, “RNK file”, or “Visualization”. ­


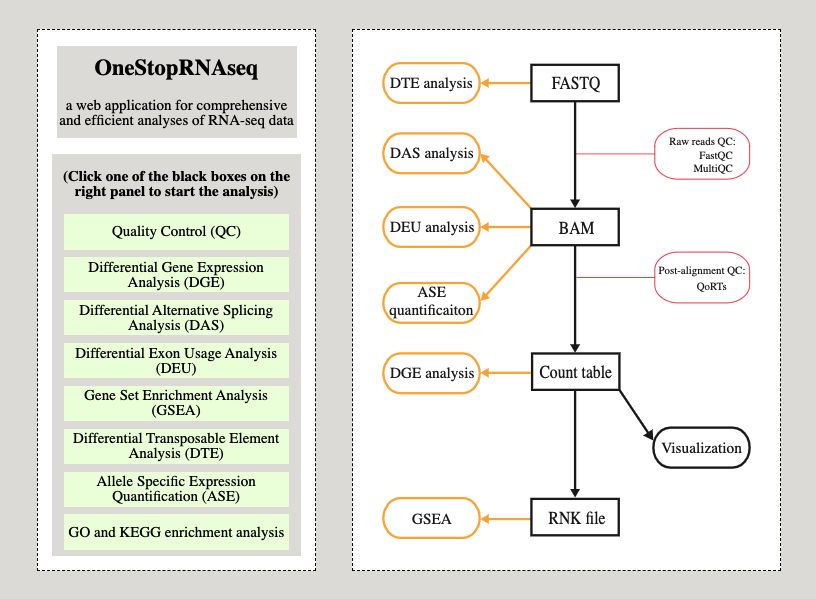


**Figure 1.** A screenshot showing the homepage of the OneStopRNAseq web application

If users choose to start the analysis with “FASTQ” files, all types of analyses will be performed, though ASE quantification requires users to provide a variant call format (VCF) file containing genotype information. If users start from “BAM” files, DTE analysis will not be performed. If users start the analysis with “Count table”, DAS will not be performed. If users start the analysis with “RNK file”, only GSEA will be performed.

In terms of the “Visualization” module, we are currently hosting DEBrowser, shiny-seq and some light yet handy online plotting tools.

**Example**

**Submit new jobs:**

In this section, we will demonstrate how to submit new jobs with “FASTQ” as the analysis entry point. As shown in Figure 2, a new study can be created by clicking the “Upload from Dropbox” or “Upload from GEO” button.


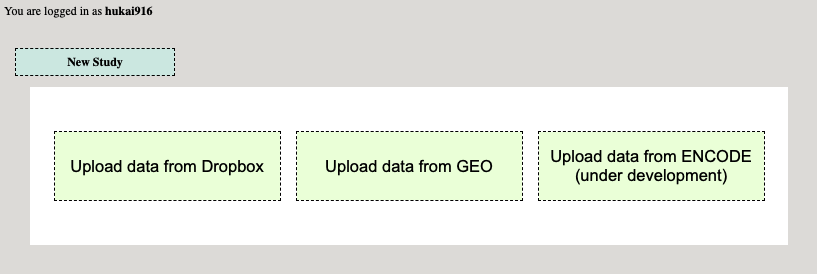


**Figure 2.** A screenshot showing the web interface for creating a new study

Once “Upload from Dropbox” button is clicked, users will be prompted to specify some basic information about their study. Click “Example 1” to see an example as shown in **Figure 3** below. Users can select multiple types of analysis. The most common combination of analyses that users are interested in are differential gene expression analysis and GSEA.

**
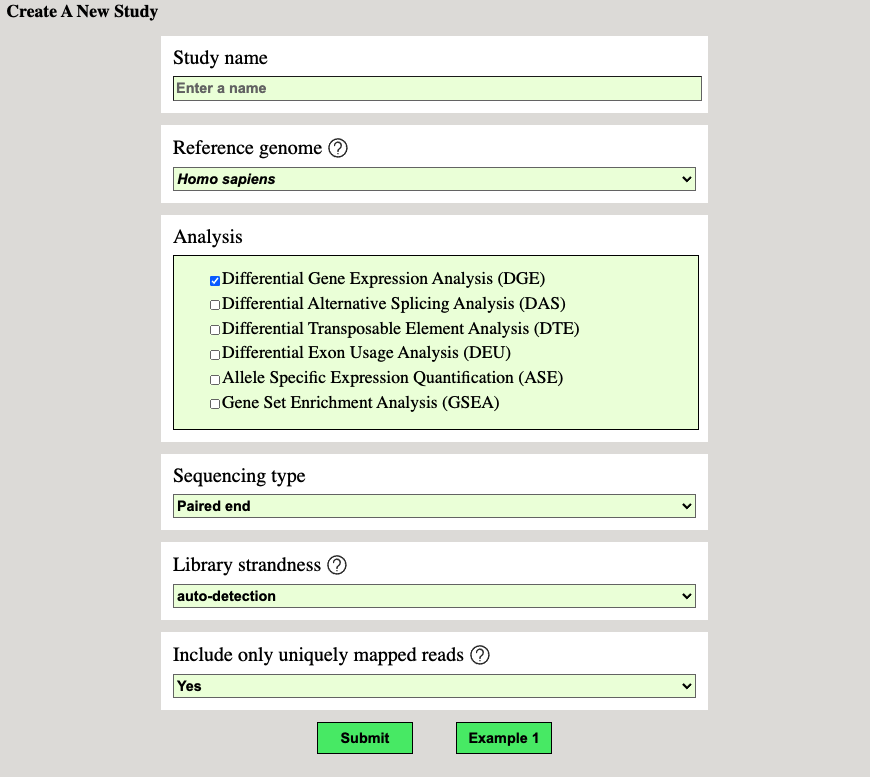
**

**Figure 3.** A screenshot showing the web interface for creating a new study.

Next, users need to provide the metadata (description of the data) and Dropbox links to the data for the study. An example is shown in **Figure 4** below. Alternatively, users can upload an Excel file prefilled with the same information.


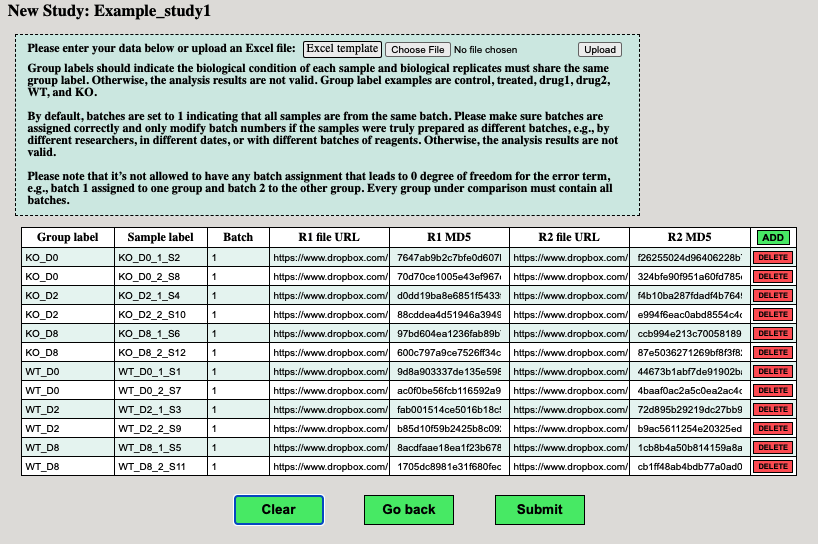


**Figure 4.** A screenshot showing the web interface to inputting metadata

After clicking the “Submit” button, users can modify the default parameter setting to fit their own needs for DEG and DAS analysis (**Figure 5**). For example, users can set the minimum log2 fold change (min_LFC) and maximum false discovery rate (max_FDR) for identifying differentially expressed genes or alternative splicing events. OneStopRNAseq is so flexible that users can make any types of contrasts/comparisons among different sample groups by clicking “Add more” button and selecting treatments to be included in group A and group B. In the results, LFC are computed as log2(groupA / groupB). The following example (Figure 5-1) shows that KO_D0, KO_D2, and KO_D8 are selected for group A, and WT_D0, WT_D2, and WT_D8 are selected for group B, which means you are interested in obtaining the main effect of KO vs WT. If there is a significant interaction between genotype and time points, then different contrasts can be easily specified as following (Figure 5-2). Select KO_D0 for group A and WT_D0 for group B to compare KO and WT at time point 0. Select KO_D2 for group A and WT_D2 for group B to compare KO and WT at time point 2. Select KO_D8 for group A and WT_D8 for group B to compare KO and WT at time point 8. Users can also set up contrasts easily to compare different time points within each genotype. Select WT_D2 for group A and WT_D0 for group B to compare time 2 and 0 for WT. Select WT_D8 for group A and WT_D0 for group B to compare time 8 and 0 for WT. Similarly, you can set up same contrasts for KO. If you are interested in genes that behaves differently in KO from time point 0 to 2 or 8 comparing to WT, then you simply select KO_D2 or KO_D8 and WT_D0 for group A and WT_D2 or WT_D8 and KO_D0 for group B.


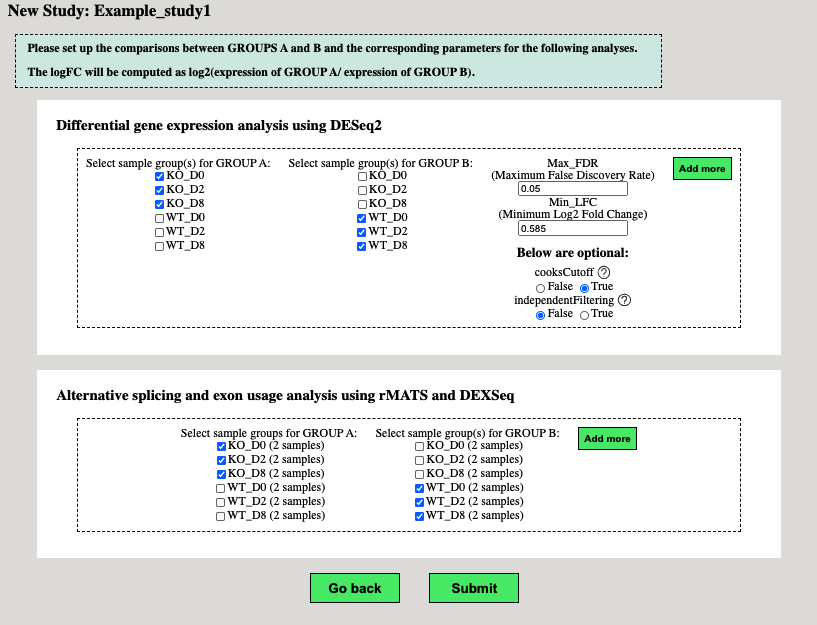


**Figure 5-1.** A screenshot showing the interface to specify analysis specific parameters and contrasts


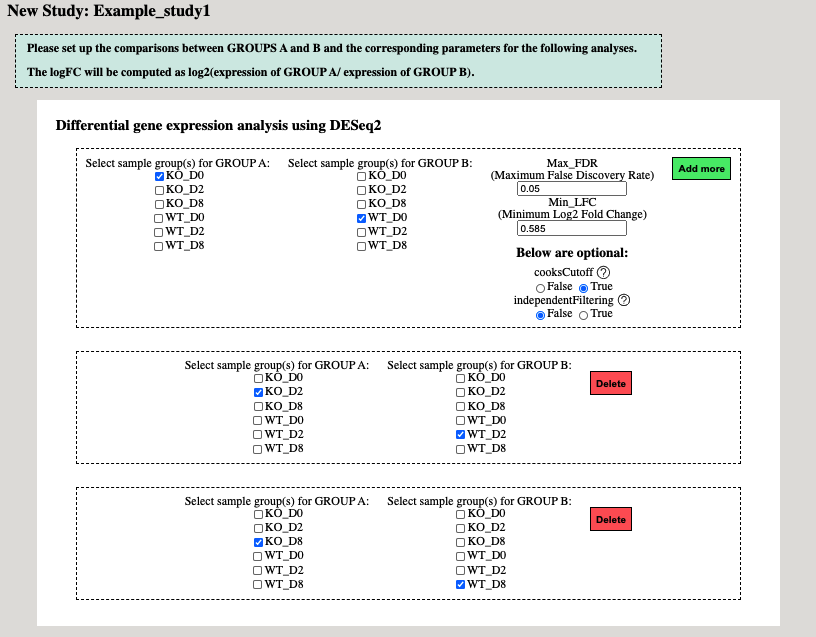


**Figure 5-2.** A screenshot showing interface to set up multiple contrasts to compare genotype effects at different time points

After clicking the “Submit” button, users will be presented a review and verification page before finalizing their submission. After the final submission, your job will be sent to our server. You will receive an email once the job is done. You can also view the analysis status by clicking the “View History” link on the right side of the top menu as detailed below.

**View history:**

Once you click on the “View History” link, all of your submitted jobs will be listed as shown in **Figure 6** below. Results can be viewed and downloaded if the “Analysis status code” starts with the number 5. A list of available status codes and their interpretation are available on the top of the analysis history. Briefly, code starting with 4 means that job is running, code starting with 5 indicates that job is finished, and the last 3 digits represent the percent of jobs successfully run. For example, code 5098 means 98% of jobs has been finished, and code 5000 means that the job has been successfully submitted with 0% finished.


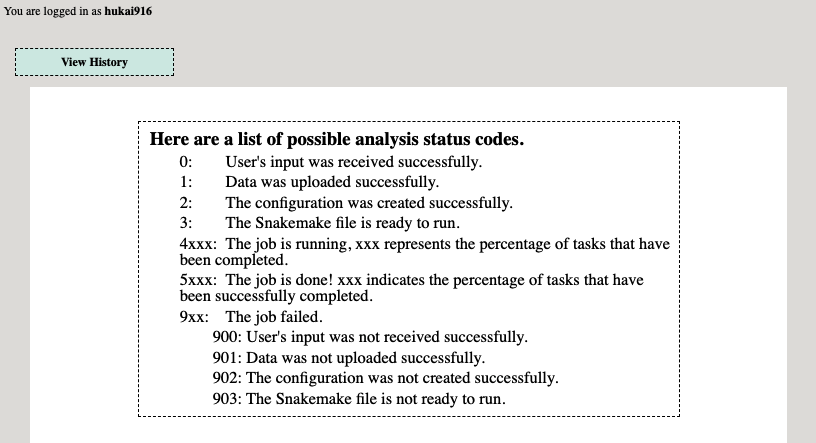

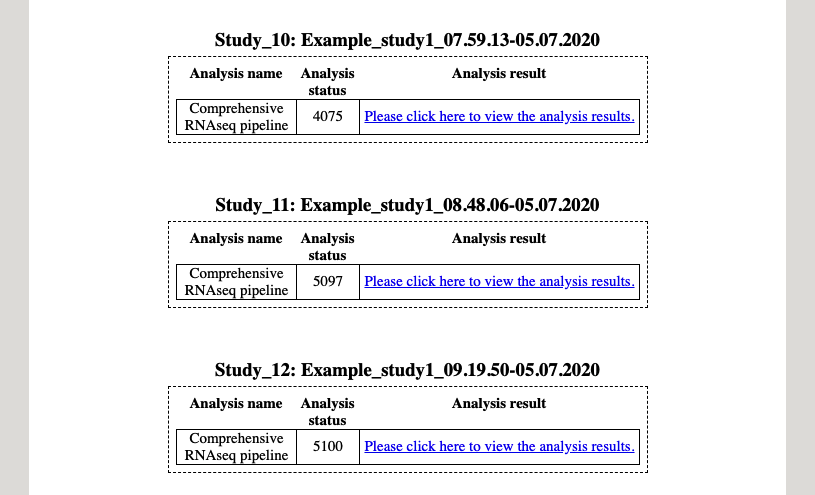


**Figure 6.** A screenshot showing the analysis history page

To further choose which type of analysis to view, please click the link below each analysis as shown in **Figure 7**.


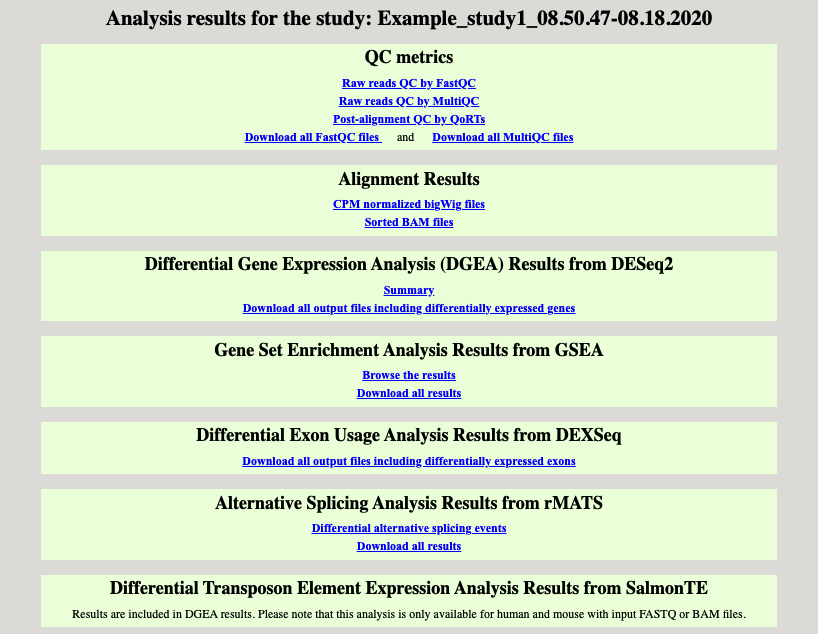


**Figure 7.** A screenshot showing the interface for reviewing analysis results

**Starting with GEO accession number**

To analyze data from GEO, users only need to provide a GEO accession number without the need to download the data to their local computer or dropbox first (**Figure 8**).


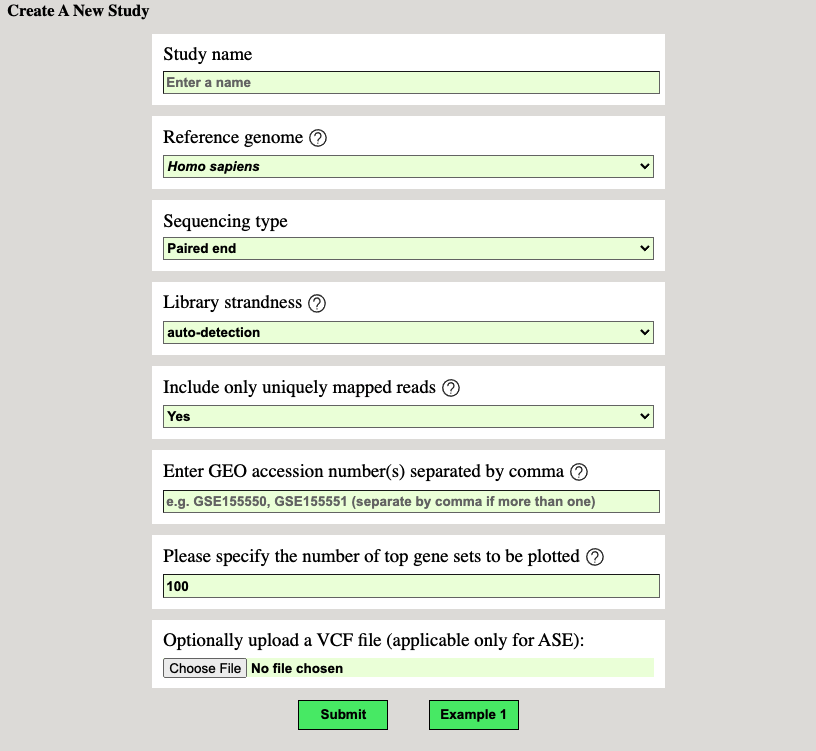


**Figure 8.** A screenshot showing the web interface for creating a new study with a GEO accession number.

After users click on the submit button, all associated sample information for the given GEO accession number will be automatically retrieved as shown in **Figure 9**. If multiple GEO accession numbers are provided, please separate them by comma. Please note that the “Group labels” are parsed out from “Sample labels” and manual corrections might be required. The “Batch” values are all set to 1 by default, users can modify them according to the experimental design. The subsequent steps are the same as starting from Dropbox links.

**
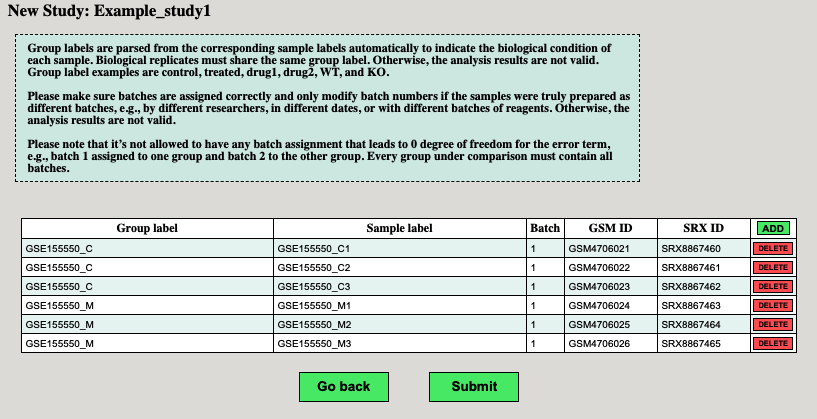
**

**Figure 9.** A screenshot showing the metadata retrieved for a given GEO accession number.

**Example – other analysis entry points:**

Instead of entering from “FASTQ” or “BAM” file, you can also choose to enter the pipeline from “Count table” or “RNK file”. Once you click “Count table” or “RNK file” on the main workflow, you will be prompted to provide required data such as “Count table” as shown in **Figure 10** . Click on the “Example file” button to download the example input files. To start analysis from “Count Table”, see below for detailed file format requirements.


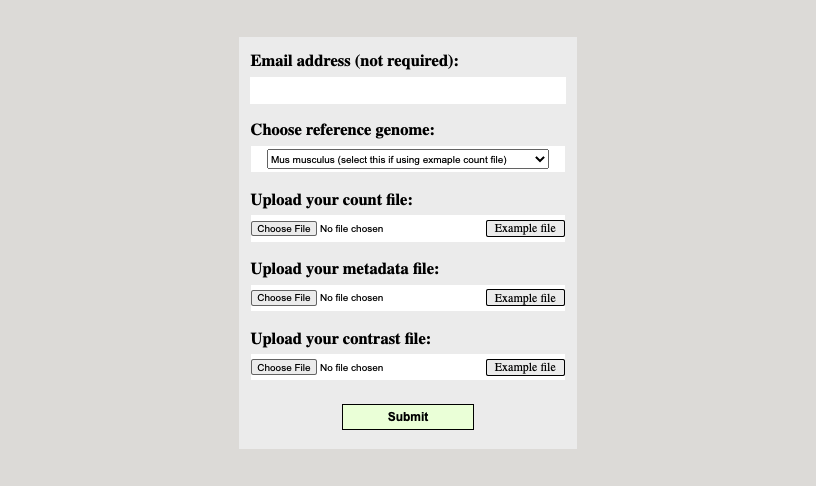


**Figure 10**. A screenshot showing the interface for starting analysis with a count file.

**Count file**: It is an Excel table with the first column as gene-id/gene-name, column two to six as additional annotation, and other columns as expression quantification as number of reads. Please note that only integers representing the raw read numbers can be used here. If you input normalized expression values, the statistics reported by DESeq2 will be invalid. If you don’t have additional annotation, column 2-6 can be left empty.

**Metadata file**: If you start the workflow from FASTQ or BAM files, metadata such as SAMPLE_LABEL, GROUP_LABEL, and BATCH information can be entered during the data submission. When you start from Count Table, you can upload an Excel file with the metadata filled in using the provided template file meta.xlsx. Please make sure that you label the samples consistently, as labels are case sensitive and different labels are considered different samples, groups, or batches by the analysis workflow. When you start from FASTQ or BAM files, ‘SAMPLE_LABEL’ should be the same as FASTQ or BAM file prefixes, as OneStopRNAseq with use this to match metadata with input files. For example, ‘SAMPLE_LABEL’ of KD_D0 should be matched with KD_D0.R1.fastq.gz and KD_D0.R2.fastq.gz for paired-end (PE) RNAseq reads, with KD_D0.fastq.gz for single-end (SE) RNAseq reads, or with KD_D0.bam for aligned BAM file.

**Contrast/Comparison file for DGE analysis**: It is an Excel file with three rows. Briefly, row 1 contains the header, row 2 contains the treatment groups, row 3 contains the control groups. The resulting log2 fold change (LFC) are calculated as log2(treatment/control). The group labels should be one of the group labels from meta.xlsx. If you need to include more than one group in the treatment or control group, just separate group labels with semicolon, as shown in the template. You can download the template Excel table contrast.de.xlsx and modify it accordingly.

**Contrast/Comparison file for DAS analysis**: This file uses the same format and specification as contrast.de.xlsx with one additional constraint, i.e., treatment group and control group need to have the same number of samples per the requirement of rMATS.

To start the analysis from “RNK file”, you will need to specify the “Rank file” as below.

**Rank file**: It is a tab-delimited file with two columns sorted by the second column in ascending order. The first column should contain the gene-symbol. The second column should be numeric that will be used to rank genes, such as LFC (log fold change), gene expression level, or even binding affinity from ChIP-seq or other experiments. Currently only human and mouse gene-symbols are supported. A template can be downloaded as ‘name1.rnk.txt’.

It is important not to include spaces in the name of any input files or sample/group labels. For sample/group labels, don’t start the label with numbers, and only alphabets, underscore, numbers are accepted.
