# Supplementary figures and images for: OneStopRNAseq: A Web Application for Comprehensive and Efficient Analyses of RNA-Seq Data
Source: Genes (Basel). 2020 Oct 2;11(10):1165. doi: 10.3390/genes11101165 (PMC7650687; doi:10.3390/genes11101165)

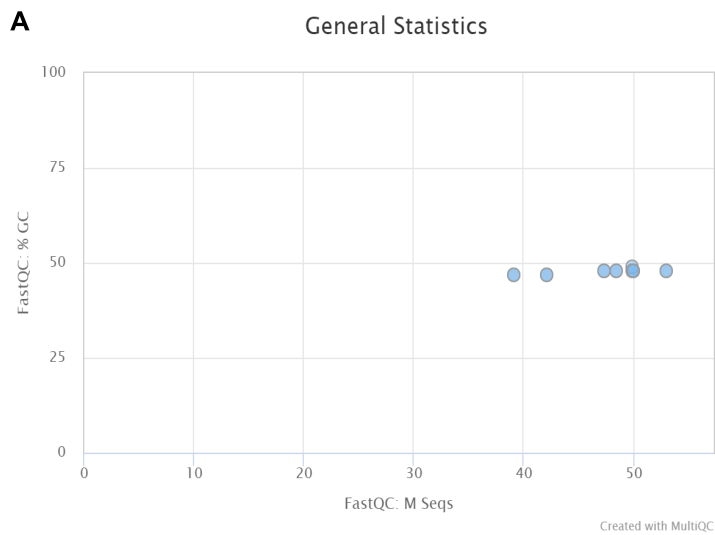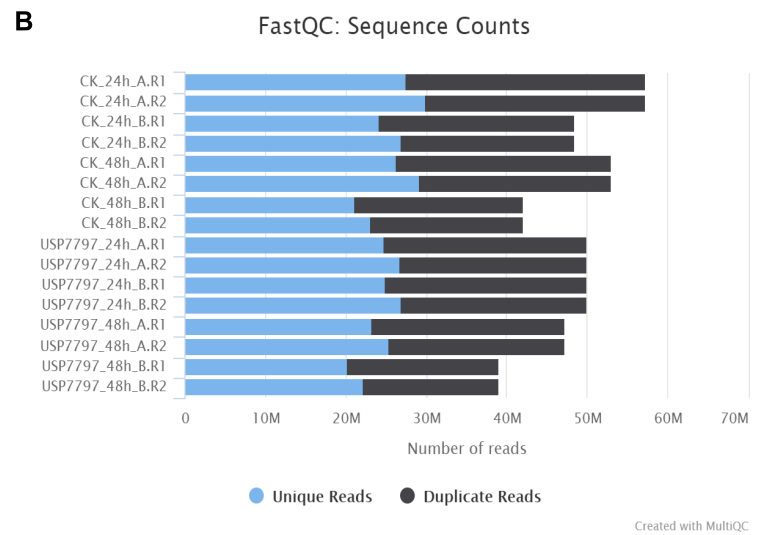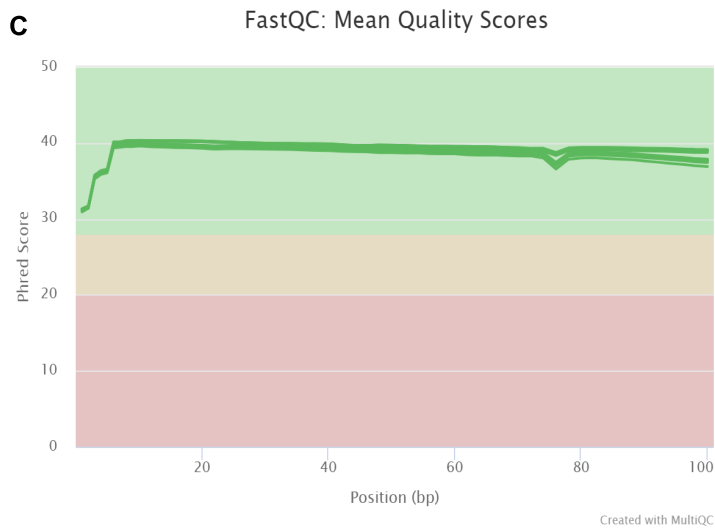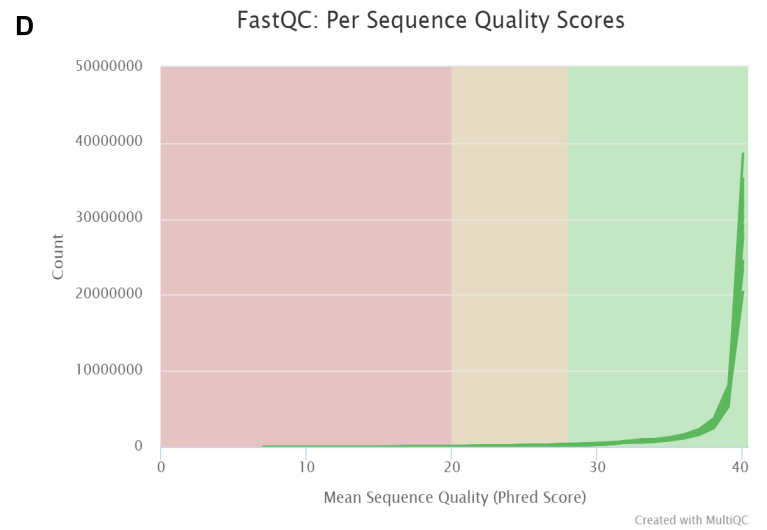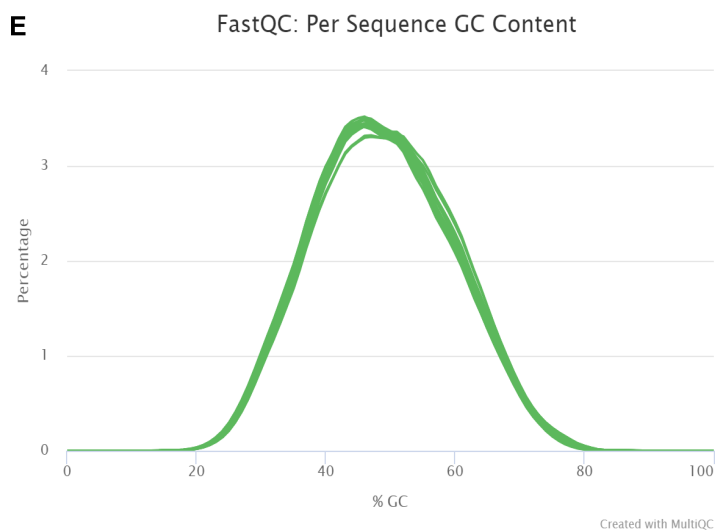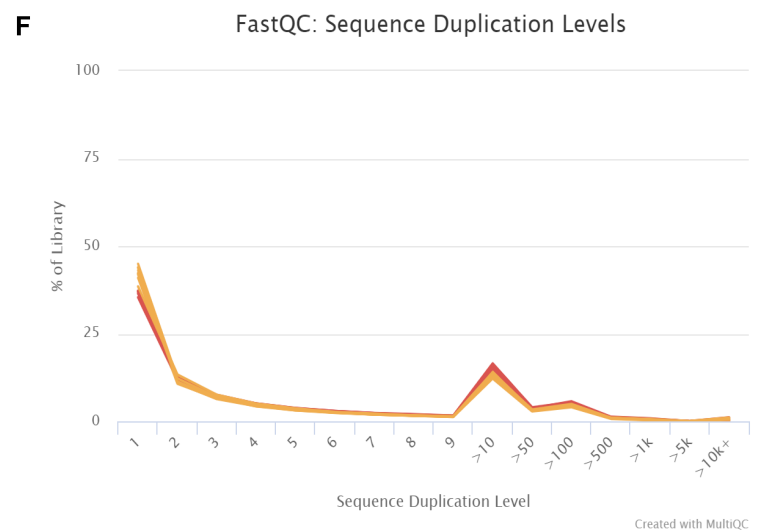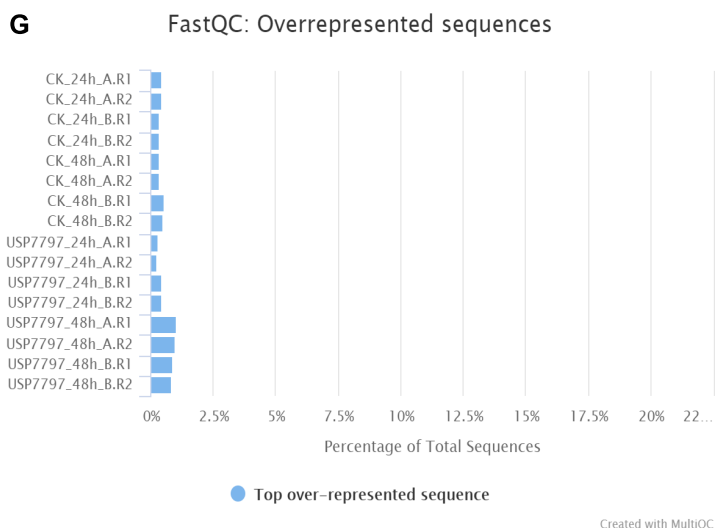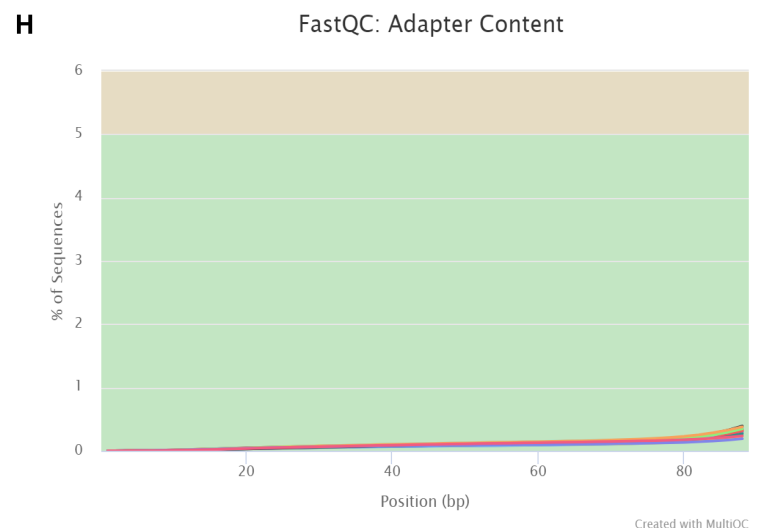

Supplement: Supplementary file 1 [file genes-11-01165-s001.zip › SupplementaryFiles/Figure S1. FASTQC.pdf]
